# An Empirical Study on the Use and Misuse of Java 8 Streams

**DOI:** 10.1007/978-3-030-45234-6_5

**Published:** 2020-03-13

**Authors:** Raffi Khatchadourian, Yiming Tang, Mehdi Bagherzadeh, Baishakhi Ray

**Affiliations:** 8 University of Paderborn, Paderborn, Germany; 9 ICREA, Open University of Catalonia, Barcelona, Spain; 10 CUNY Hunter College, New York, NY USA; 11 CUNY Graduate Center, New York, NY USA; 12 Oakland University, Rochester, MI USA; 13 Columbia University, New York, NY USA

**Keywords:** empirical studies, functional programming, Java 8, streams, multi-paradigm programming, static analysis.

## Abstract

Streaming APIs allow for big data processing of native data structures by providing MapReduce-like operations over these structures. However, unlike traditional big data systems, these data structures typically reside in shared memory accessed by multiple cores. Although popular, this emerging hybrid paradigm opens the door to possibly detrimental behavior, such as thread contention and bugs related to non-execution and non-determinism. This study explores the use and misuse of a popular streaming API, namely, Java 8 Streams. The focus is on how developers decide whether or not to run these operations sequentially or in parallel and bugs both specific and tangential to this paradigm. Our study involved analyzing 34 Java projects and 5:53 million lines of code, along with 719 manually examined code patches. Various automated, including interprocedural static analysis, and manual methodologies were employed. The results indicate that streams are pervasive, parallelization is not widely used, and performance is a crosscutting concern that accounted for the majority of fixes. We also present coincidences that both confirm and contradict the results of related studies. The study advances our understanding of streams, as well as benefits practitioners, programming language and API designers, tool developers, and educators alike.

## 1 Introduction

Streaming APIs are widely-available in today's mainstream Object-Oriented programming (MOOP) languages and platforms [5], including Scala [14], JavaScript [44], C# [33], F# [47], Java [39], and Android [27]. These APIs allow for "big data"-style processing of *native* data structures by incorporating MapReduce-like [10] operations. A "sum of even squares" example in Java, where a `stream` of numbers is derived from a `list`, `filter`ed for evens, `map`ped to its squared, and summed [5] is: `list.stream().filter(x -> x % 2 == 0).map(x -> x * x).sum()`.

Traditional big data systems, for which MapReduce is a popular backbone [3], minimize the complexity of writing massively distributed programs by facilitating processing on multiple nodes using succinct functional-like constructs. This makes writing parallel code easier, as writing such code can be difficult due to possible data races, thread interference, and contention [1,4,28]. The code above, e.g., can execute in parallel simply by replacing `stream()` with `parallelStream()`.

However, unlike traditional big data systems, data structures processed by streaming APIs like Java 8 Streams typically reside in shared memory accessed by multiple cores. Therefore, issues may arise from the close intimacy between shared memory and the operations being performed, especially for developers not previously familiar with functional programming. Streams are not just an API but rather an emerging, hybrid paradigm. To obtain the expressiveness, speed, and parallelism that streams have to offer, developers must adopt the paradigm as well as the API [6, Ch. 7]. This requires determining whether running stream code in parallel yields an efficient yet interference-free program [24] and ensuring that no operations on different threads interleave [42].

Despite the benefits [53, Ch. 1], misusing streams may result in detrimental behavior, and the ~4K questions related to streams on Stack Overflow [48], of which ~5% remain unanswered, suggest that there is ample confusion surrounding the topic. Bugs related to thread contention (due to $\lambda$-expressions, i.e., units of computation, side-effects, buffering), non-execution (due to deferred execution), non-determinism (due to non-deterministic operations), operation sequencing (ordering of stream operations), and data ordering (ordering of stream data) can lead to programs that undermine concurrency, underperform, are incorrect, and are inefficient. Worse yet, these problems may increase over time as streams rise in popularity, with Mazinanian et al. [32] finding a two-fold increasing trend in the adoption of $\lambda$-expressions, an essential part of streams.

This study explores the use and misuse of a popular and representative streaming API, namely, Java 8 Streams. We set out to understand the *usage* and *bug* patterns involving streams in real software. Particularly, we are interested in discovering (i) how developers decide whether to run streams *sequentially* or in *parallel*, (ii) common stream *operations*, (iii) common stream *attributes* and whether they are amenable to safe and efficient parallelization, (iv) *bugs* both specific and tangential to streams, (v) how often *incorrect* stream APIs were used, and (vi) how often stream APIs were *misused* and in which ways?

Knowing the kinds of bugs typically associated with streams can, e.g., help improve (automated) bug detection. Being aware of the typical usage patterns of streams can, e.g., improve code completion in IDEs. In general, the results (i) advance our understanding of this emerging hybrid paradigm, (ii) provide feedback to language and API designers for future API versions, (iii) help tool designers comprehend the struggles developers have with streams, (iv) propose preliminary *best practices* and *anti-patterns* for practitioners to use streaming APIs effectively, (v) and assist educators in teaching streaming APIs.

We analyzed 34 Java projects and 5.53 million lines of source code (SLOC), along with 140,446 code patches (git commits), of which 719 were manually

**Listing 1** Snippet of `Widget` collection processing using Java 8 streams [24,39].

```
1  Collection<Widget> unorderedWidgets = new HashSet<>(); // populate ...
2  Collection<Widget> orderedWidgets = new ArrayList<>(); // populate ...
3  List<Widget> sortedWidgets = unorderedWidgets.stream()
4      .sorted(Comparator.comparing(Widget::getWeight)).collect(Collectors.toList());
5  // collect weights over 43.2 into a set in parallel.
6  Set<Double> heavyWidgetWeightSet = orderedWidgets.parallelStream().map(Widget::getWeight)
7      .filter(w -> w > 43.2).collect(Collectors.toSet());
8  // sequentially collect into a list, skipping first 1000.
9  List<Widget> skippedWidgetList = orderedWidgets.stream().skip(1000)
10     .collect(Collectors.toList());
```

examined. The methodologies varied depending on the research questions and encompassed both automated, including interprocedural static analysis, and manual processes aided by automated software repository mining. Our study indicates that (i) streams have become widely used since their inception in 2014, (ii) developers tend to reduce streams back to iterative-style collections, favor simplistic, linear reductions, and prefer deterministic operations, (iii) stream parallelization is not widely used, yet streams tend not to have side-effects, (iv) performance is the largest category of stream bugs and is crosscutting.

This work makes the following contributions:

**Stream usages patterns** A large-scale analysis of stream and collector method calls and an interprocedural static analysis on 1.65 million lines source code is performed, reporting on attributes essential to efficient parallel execution.

**Stream bug hierarchical taxonomy** From the 719 git patches from 22 projects manually examined using 140 identifying keywords, we build a rich hierarchical, crosscutting taxonomy of common stream bugs and fixes.

**Best practices and anti-patterns** We propose preliminary best practices and anti-patterns of using streams in particular contexts from our statistical results as well as an in-depth analysis of first-hand conversations with developers.

## 2 Motivating Example and Conceptual Background

Lst. 1 portrays code that uses the Java 8 Stream API to process collections of `Widgets` (class not shown) with `colors` and `weights`. A `Collection` of `Widgets` is declared (line 1) that does not maintain element ordering as `HashSet` does not support it [38]. Note that ordering is dependent on the run time type.

A `stream` (a view representing element sequences supporting MapReduce-style operations) of `unorderedWidgets` is created on line 3. It is sequential, i.e., its operations will execute serially. Streams may also have an *encounter order* that may depend on its source. Here, it is unordered since `HashSets` are unordered.

On line 4, the stream is `sorted` by the corresponding *intermediate* operation, the result of which is a stream with the encounter order rearranged. `Widget::getWeight` is a method *reference* denoting the comparison scheme. Intermediate operations are deferred until a *terminal* operation is executed like `collect()` (line 4). The `collect()` operation is a (mutable) reduction that aggregates results of prior intermediate operations into a given `Collector`. In this case, it is one that yields a `List`. The result is a `Widget List` sorted by `weight`.

To potentially improve performance, this stream's "pipeline" (sequence of operations) may be executed in parallel. Note, however, that had the stream been *ordered*, running the pipeline in parallel may result in worse performance due to the multiple passes or data buffering required by *stateful* intermediate operations (SIOs) like `sorted()`. Because the stream is *unordered*, the reduction can be done more efficiently as the run time can use divide-and-conquer [39].

In contrast, line 2 instantiates an `ArrayList`, which maintains element ordering. Furthermore, a parallel stream is derived from this collection (line 6), with each `Widget` mapped to its weight, each weighted filtered (line 7), and the results `collect`ed into a `Set`. Unlike the previous example, however, no optimizations are available here as an SIO is not included in the pipeline and, as such, the parallel computation does not incur possible performance degradation.

Lines 9–10 create a list of `Widget`s gathered by (sequentially) skipping the first thousand from `orderedWidgets`. Like `sorted()`, `skip()` is also an SIO. Unlike the previous example, executing this pipeline in parallel could be counterproductive because the stream is ordered. It may be possible to unorder the stream (via `unordered()`) so that its pipeline would be more amenable to parallelization. In this situation, however, unordering could alter semantics as the data is assembled into a structure maintaining ordering. As such, the stream *correctly* executes sequentially as element ordering must be preserved.

This simplified example demonstrates that using streams effectively is not always straight-forward and can require complex (and interprocedural due to aliasing) analysis. It necessitates a thorough understanding of API intricacies, a problem that can be compounded in more extensive programs. As streaming APIs become more pervasive, it would be extremely valuable to MOOP developers not familiar with functional programming if statistical insight can be given on how best to use streams efficiently and how to avoid common bugs.

## 3 Study Subjects

At the core of our study is 34 open source Java projects that use streams. They vary widely in their domain and application, as well as size and popularity. All the subjects have their sources publicly available on GitHub and include popular libraries, frameworks, and applications. Many subjects were selected from previous studies [20,21,22,24], others because they contained relatively diverse stream operations and exhibited non-trivial metrics, including stars, forks, and number of collaborators. It was necessary to use different subjects for different parts of the study due to the computationally intensive nature of some of the experiments. For such experiments, subjects were chosen so that the analysis could be completed in a reasonable time period with reasonable resources.

## 4 Stream Characteristics

We explore the typical usage patterns of streams, including the frequency of parallel vs. sequential streams and amenability to safe and efficient parallelism, by examining stream characteristics. This has important implications for understanding the use of this incredibly expressive and powerful language feature. It also offers insight into developers' perceived risks concerning parallel streams.

**Table 1.** Stream characteristics.

| subject | KLOC | age | eps | k | str | seq | para | ord | unord | se | SIO |
|---|---|---|---|---|---|---|---|---|---|---|---|
| bootique | 4.91 | 4.18 | 362 | 4 | 14 | 14 | 0 | 11 | 3 | 4 | 0 |
| cryptomator | 7.99 | 6.05 | 148 | 3 | 12 | 12 | 0 | 11 | 1 | 2 | 0 |
| dari | 64.86 | 5.43 | 3 | 2 | 18 | 18 | 0 | 15 | 3 | 0 | 0 |
| elasticsearch | 585.71 | 10.03 | 78 | 6 | 210 | 210 | 0 | 165 | 45 | 10 | 0 |
| htm.java | 41.14 | 4.53 | 21 | 4 | 190 | 188 | 2 | 189 | 1 | 22 | 5 |
| JabRef | 138.83 | 16.36 | 3,064 | 2 | 301 | 290 | 11 | 239 | 62 | 9 | 0 |
| JacpFX | 23.79 | 4.71 | 195 | 4 | 12 | 12 | 0 | 9 | 3 | 1 | 0 |
| jdp* | 19.96 | 5.53 | 25 | 4 | 38 | 38 | 0 | 35 | 3 | 11 | 1 |
| jdk8-exp* | 3.43 | 6.35 | 34 | 4 | 49 | 49 | 0 | 47 | 2 | 5 | 0 |
| jetty | 354.48 | 10.93 | 106 | 4 | 57 | 57 | 0 | 47 | 10 | 8 | 0 |
| JetUML | 20.95 | 5.09 | 660 | 2 | 7 | 7 | 0 | 4 | 3 | 0 | 0 |
| jOOQ | 154.01 | 8.58 | 43 | 4 | 23 | 23 | 0 | 22 | 1 | 2 | 0 |
| koral | 7.13 | 3.47 | 51 | 3 | 8 | 8 | 0 | 8 | 0 | 0 | 0 |
| monads | 1.01 | 0.01 | 47 | 2 | 3 | 3 | 0 | 3 | 0 | 0 | 0 |
| retrolambda | 5.14 | 6.52 | 1 | 4 | 11 | 11 | 0 | 8 | 3 | 0 | 0 |
| spring* | 188.46 | 11.62 | 5,981 | 4 | 61 | 61 | 0 | 60 | 1 | 21 | 0 |
| streamql | 4.01 | 0.01 | 92 | 2 | 22 | 22 | 0 | 22 | 0 | 2 | 18 |
| threeten* | 27.53 | 7.01 | 36 | 2 | 2 | 2 | 0 | 2 | 0 | 0 | 0 |
| Total | 1,653.35 | 116.40 | 11,047 | 6 | 1,038 | 1,025 | 13 | 897 | 141 | 97 | 24 |

* jdp is java-design-patterns, jdk8-exp is jdk8-experiments, spring is a portion of spring-framework, and threeten is threeten-extra.

### 4.1 Methodology

For this part of the study, we examined 18 projects that use streams,[5] spanning ∼1.65 million lines of Java source code. The subjects are depicted in tab. 1. Column **KLOC** corresponds to thousands of source lines of code, which ranges from ∼1K for monads to ∼586K for elasticsearch. Column **age** is the age of the subject project in years, averaging 6.47 years per subject. Column **str** is the total number of streams analyzed. The remaining columns are discussed in § 4.2.

**Stream Pipeline Tracking** Several factors contribute to determining stream attributes. First, streams are typically derived from a source (e.g., a collection) and take on its characteristics (e.g., ordering), as seen in lst. 1. There are several ways to create streams, including being derived from `Collections`, being created from arrays (e.g., `Arrays.stream()`), and via static factory methods (e.g., `IntStream.range()`). Second, stream attributes can change by the invocation of various intermediate operations in the building of the stream pipeline. Such attributes must be tracked, as it is possible to have arbitrary assignments of stream references to variables, as well as be data-dependent.

Our study involved tracking streams and their attributes (i.e., state) using a series of labeled transition systems (LTSs). The LTSs are fed into the static analysis portion of a refactoring tool [23] based on typestate analysis [16,49]. Stream pipelines are tracked and stream state when a terminal operation is issued is determined by the tool. Typestate analysis is a program analysis that augments the type system with "state" information and has been traditionally used for prevention of program errors such as those related to resource usage. It works by assigning each variable an initial (⊥) state. Then, method calls transition the object's state. States are represented by a lattice and possible transitions

[5] Recall from § 3 that it was necessary to use different subjects for different parts of the study due to the computationally intensive nature of some of the experiments.

are represented by LTSs. If each method call sequence on the receiver does not eventually transition the object back to the ⊥ state, the object may be left in a nonsensical state, indicating the potential presence of a bug.

The LTSs for execution mode and ordering work as follows. The state ⊥ is a phantom initial state immediately before stream creation. Different stream creation methods may transition the newly created stream to one that is either sequential or parallel or ordered or unordered. The transition continues for each invoked intermediate operation and ends with a terminal operation.

Since the analysis is focused on client-side analysis of stream APIs, the call graph is constructed using a $k$-CFA, where $k$ is the call string length. It is an analysis parameter, with $k = 2$ being the default, as it is the minimum $k$ needed to consider client-code, for methods returning streams and $k = 1$ elsewhere. The refactoring tool includes heuristics for determining sufficient and tractable $k$.

**Counting Streams** Since stream attributes are control flow sensitive, the streams studied must be in the control flow of entry points. For non-library subjects, all main methods were chosen, otherwise, all unit tests were chosen.

Streams are counted as follows. First, every *syntactic* stream is counted, i.e., every allocation site. Streams in the control flow of the program starting from an entry point transition according to the LTSs. If a stream is not in the control flow, it is still counted but it remains at the state following ⊥. This way, more information about various stream attributes is available for the study as we do not need control flow to determine the state following ⊥.

**Side-effects and Stateful Intermediate Operations** Stream side-effects are determined using a ModRef analysis on stream operation parameters ($\lambda$-expressions) using WALA [52]. SIOs are obtained from the documentation [39].

### 4.2 Results

Tab. 1 illustrates our findings on stream characteristics. Column **eps** is the number of entry points. Column **k** is the maximum $k$ value used (see § 4.1). Columns **seq** and **para** correspond to the number of *sequential* and *parallel* streams, respectively. Column **ord** is the number of streams that are *ordered*, i.e., those whose operations must maintain an encounter order, which can be detrimental to efficient parallel performance, while column **unord** is the number *unordered* streams. Column **se** is the number of stream pipelines that include *side-effects*, which may induce race conditions. Finally, column **SIO** is the number of pipelines that include *stateful intermediate operations*, which may also be detrimental to efficient parallel performance.

### 4.3 Discussion

Parallel streams are not popular (1.25%) despite their ease-of-use. Although Nielebock et al. [36] did not consider $\lambda$-expressions in stream contexts, this confirms that their findings extend into stream contexts. It may also coincide with the finding of Lu et al. [28], i.e., that developers tend to "think" sequentially.

| ***Finding 1***: Stream parallelization is not widely used. |
|---|

When considering using parallel streams, it may also be important to consider the *context*. For example, many server applications deal with thread pools that span the JVM, and developers may be leery of the interactions of such pools with the underlying stream parallelization run time system. We found this to be the case with several pull requests [15,45] that were issued by Khatchadourian et al. [24] as part of their refactoring evaluation to introduce parallel streams into existing projects. It may also be the case that the locations where streams operate are already fast enough or do not process significant amounts of data [7,30]. In fact, Naftalin [35, Ch. 6] found that there is a particular threshold in data size that must be reached to compensate for overhead incurred by parallel stream processing. Lastly, developers pointed us to several blog articles [54,59] expressing that parallel streams could be problematic under certain conditions.

There were, however, two projects that use parallel streams. Particularly, JabRef used the most parallel streams at 11. We conjecture that JabRef's use of parallel streams may stem from its status as a desktop application. Such applications typically are not managed by application containers and thus may not utilize global thread pools as in more traditional server applications.

Many streams are ordered (86.42%), which can prevent optimal performance of parallel streams under certain conditions [24,35,40]. Thus, even if streams were run in parallel, they may not reap all of the benefits. This extends the findings of Nielebock et al. [36] that $\lambda$-expressions do not appear in contexts amenable to parallelization to streams for the case of ordering. Streams may still be amenable to parallelization, as § 5.2 shows that many streams are traversed using API that *ignores* ordering (e.g., `forEach()` vs. `forEachOrdered()`).

***Finding 2***: Streams are largely *ordered*, possibly hindering parallelism.

That only ∼10% of streams have side-effects and only 2.31% have SIOs contradict the findings of Nielebock et al. [36] in the context of streams. This suggests that streams may run efficiently in parallel as, although they are largely ordered, they include minimal side-effects and SIOs. streamql had the most streams with SIOs (18/22), which may be due to its querying features using aggregate operations that are manifested as SIOs in the Java 8 Streaming API (e.g., `distinct()`).

***Finding 3***: Streams tend *not* to have side-effects.

## 5 Stream Usage

We discover the common operations on streams and the underlying reasons by examining stream method calls. This has important implications in understanding how streams are used, and studying language feature usage has been shown to be beneficial [11,43]. It provides valuable insight to programming language API designers and tool-support engineers on where to focus their evaluation efforts. We may also comprehend contexts where developers struggle with using streams.

### 5.1 Methodology

We examined 34 projects that use streams, spanning ∼5.53 million lines of source code. To find method calls, we parsed ASTs with source-symbol bindings using the Eclipse Java Developer Tools (JDT) [12]. Then, method invocation nodes were extracted whose compile-time targets are declared in types residing in the `java.util.stream` package. This includes types such as `Streams` and `Collectors`.

While stream creation is interesting and a topic for future work, our focus is on operations *on* streams as our scope is stream *usage*. We also combined methods with similar functionalities, e.g., `mapToLong()` with `map()` but not `forEach()` and `forEachOrdered()`. Additionally, only the method name is presented, resulting in a comparison of methods from both streams and collectors. The type is clear from the method name (e.g., `map()` is for `Streams`, while `groupingBy()` is for `Collectors`). We then proceeded to count the number of method calls in each project.

### 5.2 Results

Fig. 1 depicts the result of our analysis.[6] A full table is available in our dataset [25]. The horizontal axis lists the method name, and the legend depicts projects analyzed. The chart is sorted by the total number of calls in descending order. Calls per project range from 4 for threeten-extra to 4,635 for cyclops. Calls per method range from 2 for `characteristics()`, which returns stream attributes such as whether it is ordered or parallel, and 3,161 for `toList()`.

### 5.3 Discussion

The number of method calls in fig. 1 is substantial. There are 14,536 calls to methods operating on streams in 34 projects. This is impressive considering that Android, which uses the Java syntax, did not adopt streams immediately.

It is not surprising that the four most used stream methods are `toList()`, `collect()`, `map()`, and `filter()`, as these are the core MapReduce data transformation operations. `collect()` is a specialized reduction that reduces to a non-scalar type (e.g., a map) as opposed to the traditional scalar type. The `toList()` method is a static method of `Collectors`, which are pre-made reductions, in this case, to an `ArrayList`. This informs the `collect()` operation of the non-scalar type to use. It is peculiar that there are more calls to `toList()` than `collect()`. This is due to cyclops. We conjecture that it has some unorthodox usages of `Collectors` as it is a platform for writing functional-style programs in Java $\geq 8$ [2].

That `collect()` and `toList()`, along with other terminal operations such as `forEach()`, `iterator()`, `toSet()`, and `toArray()`, appear towards the top to the list suggest that, although developers are writing functional-style code to process data in a "big data" processing style, they are not staying there. Instead, they are "bridging" back to imperative-style code, either by collecting data into imperative-style collections or processing the data further iteratively.

There can be various reasons for this, such as unfamiliarity with functional programming, the need to introduce side-effects, or the need to interoperate with legacy code. Further investigation is necessary, yet, Nielebock et al. [36] mention that developers tend to introduce side-effects into $\lambda$-expressions, which is related.

> ***Finding 4***: Although stream usage is high, developers tend to reduce streams back to iterative-style collections.

We infer that developers tend to favor more simplistic (linear) rather than more specialized (higher-dimensionality) non-scalar reductions. It is surprising that more of the advanced reductions, such as those that return maps (e.g., `toMap()`,

[6] Similar conclusions hold when normalizing with subject KLOC.

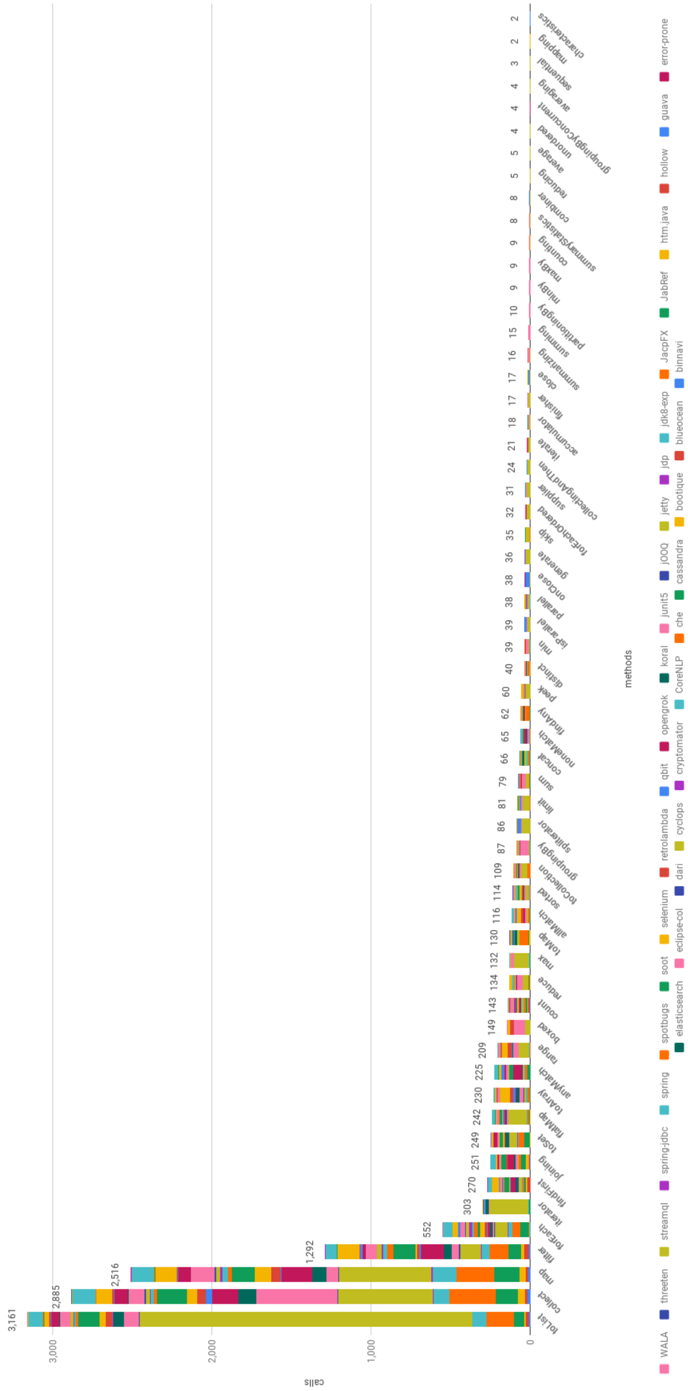

**Fig. 1.** Stream method calls

`groupingBy()`) are not used more frequently as these are highly expressive operations that can save substantial amounts of imperative-style code. For example, one may group `Widgets` by their `Color` as `Map<Color, List<Widget>> widgetsByColor = widgets.stream().collect(Collectors.groupingBy(Widget::getColor))`. Although these advanced reductions are powerful and expressive, developers may be leery of using them, perhaps due to unfamiliarity or risk adversement. This motivates future tools that refactor to uses of advanced reductions to save developers time and effort while possibly mitigating errors.

***Finding 5***: Developers favor simplistic, linear reductions.

Another powerful stream feature is its non-determinism. For instance, `findAny()` returns any stream element. However, this operation has only 62 calls, while its deterministic counterpart, `findFirst()`, has 270, suggesting that developers tend to favor determinism. Yet, in contrast, developers overwhelmingly favor the *non-deterministic* `forEach()` operation (552) over the *deterministic* `forEachOrdered()` (32). We conjecture that although `forEach()` does not guarantee a particular ordering [41], in practice, since developers are inclined to use sequential over parallel streams, as suggested by § 4 and mirrored by Nielebock et al. [36] in terms of $\lambda$-expressions, the difference does not play out.

It could also be that traversal order is largely unimportant for many streams. This is curious because, as demonstrated in § 4, the majority of streams are *ordered*, an attribute detrimental to efficient parallelism [24,35,40]. As such, there may exist opportunities to alleviate the burden of stream ordering maintenance to make parallel streams more efficient. It may also entice developers to use more parallel streams as the performance gains may be significant.

***Finding 6***: Developers prefer deterministic operations.

Lastly, there is a minimal amount of calls to parallel stream APIs. Of particular concern is that there are only 4 calls to `groupingByConcurrent()` in contrast to the 87 calls to `groupingBy()`. This suggests that either advanced reductions to maps are not being used on parallel streams or that they are not used safely as the concurrent version provides synchronization [37]. Furthermore, not using `groupingByConcurrent()` on a parallel stream may produce inefficient results [40].

## 6 Stream Misuses

This section is focused on discovering stream bug patterns. We are interested in bugs both specific and tangential to streams, i.e., bugs that occur in stream contexts. Understanding this can, e.g., help improve (automated) bug detection and other tool-support for writing optimal stream code. We may also begin to understand the kinds of errors developers make with streams, which may positively influence how future API and language feature versions are implemented.

### 6.1 Methodology

Here, we explore 22 projects that use streams, comprising ∼4.68 million lines of source code and 140,446 git commits.[7] Tab. 2 summarizes the subjects used.

[7] Recall from § 3 that it was necessary to use different subjects for different parts of the study due to the computationally intensive nature of some of the experiments.

**Table 2.** Studied subjects.

| subject | KLOC | studied periods | cmts | kws | exe |
|---|---|---|---|---|---|
| binnavi | 328.28 | 2015-08-19 to 2019-07-17 | 286 | 4 | 4 |
| blueocean-plugin | 49.70 | 2016-01-23 to 2019-07-24 | 4,043 | 118 | 25 |
| bootique | 15.47 | 2015-12-10 to 2019-08-08 | 1,106 | 5 | 5 |
| che | 189.24 | 2016-02-11 to 2019-08-19 | 8,093 | 75 | 75 |
| cryptomator | 9.83 | 2014-02-01 to 2019-08-08 | 1,443 | 50 | 10 |
| dari | 72.46 | 2012-09-26 to 2018-03-02 | 2,466 | 18 | 6 |
| eclipse.jdt.core | 1,527.89 | 2001-06-05 to 2019-08-07 | 24,085 | 234 | 106 |
| eclipse.jdt.ui | 712.91 | 2001-05-02 to 2019-08-09 | 28,136 | 149 | 32 |
| error-prone | 165.85 | 2011-09-14 to 2019-08-15 | 3,893 | 71 | 71 |
| guava | 393.47 | 2009-06-18 to 2019-08-15 | 5,031 | 36 | 36 |
| htm.java | 41.63 | 2014-08-09 to 2019-02-19 | 1,507 | 40 | 1 |
| JacpFX | 24.06 | 2013-08-12 to 2018-04-27 | 365 | 37 | 14 |
| jdk8-experiments | 3.47 | 2013-08-03 to 2018-03-10 | 8 | 1 | 1 |
| java-design-patterns | 33.52 | 2014-08-09 to 2019-07-31 | 2,192 | 37 | 12 |
| jetty | 400.26 | 2009-03-16 to 2019-08-02 | 17,051 | 835 | 219 |
| jOOQ | 184.25 | 2011-07-24 to 2019-07-31 | 7,508 | 94 | 4 |
| qbit | 52.27 | 2014-08-25 to 2018-01-18 | 1,717 | 65 | 9 |
| retrolambda | 5.10 | 2013-07-20 to 2018-11-30 | 522 | 17 | 4 |
| selenium | 234.12 | 2004-11-03 to 2019-08-09 | 24,145 | 114 | 57 |
| streamql | 4.26 | 2014-04-27 to 2014-04-29 | 27 | 2 | 2 |
| threeten-extra | 31.26 | 2012-11-17 to 2019-07-14 | 559 | 28 | 2 |
| WALA | 203.84 | 2006-11-22 to 2019-07-24 | 6,263 | 52 | 24 |
| Total | 4,683.12 | | 140,446 | 2,082 | 719 |

To find changesets (patches) corresponding to stream fixes, we compiled 140 keywords from the API documentation [39] that match stream operations and related method names from the `java.util.stream` package. We then randomly selected a subset of these commits whose changesets included these keywords and were likely to be bug fixes to manually examine.

**Commit Mining** To discover commits that had changesets including stream API keywords, we used `gitcproc` [9], a tool for processing and classifying git commits, which has been used in previous work [17,50]. Due to the keyword-based search used, not all of the examined commits pertained to streams (e.g., "map" has a broad range of applications outside of streams). To mitigate this, we focused more on keywords that were specific to stream contexts, e.g., "Collector." Also to reduce false positives, we only considered commits after the Java 8 release date of March 18, 2014, which is when streams were introduced.

**Finding Bug Fixes** We used a feature of `gitcproc` that uses heuristics based on commit log messages to identify commits that are bug fixes. Natural language processing (NLP) is used to determine which commits fall in this category. This helps us to focus on the likely bug-fix commits for further manual examination.

Next, the authors manually examine these commits to determine if the commits were indeed related to stream-related bugs. Three of the authors are software engineering and programming language professors with extensive expertise in streaming and parallel systems, concurrent systems, and empirical software engineering. The authors also have several years of industrial experience working as software engineers. As the authors did not always have expertise in the subject domains, only changes where a bug fix was extremely likely were marked as such. The authors also used commit comments and referenced bug databases to ascertain whether a change was a bug fix. This is a common practice [8,26,28].

**Table 3.** Stream bug/patch category legend.

| name | description | acronym |
|---|---|---|
| Bounds | Incorrect/Missing Bounds Check | BC |
| Exceptions | Incorrect/Missing Exception Handling | EH |
| Other | Other change (e.g., syntax, refactoring) | Other |
| Perf | Poor Performance | PP |
| Concur | Concurrency Issue | CI |
| Stream Source | Incorrect/Missing Stream Source | SS |
| Intermediate Operations | Incorrect/Missing Intermediate Operations | IO |
| Data Ordering | Incorrect Data Ordering | DO |
| Operation Sequencing | Incorrect Operation Sequencing | OS |
| Filter Operations | Incorrect/Missing Filter Operations | FO |
| Map Operations | Incorrect/Missing Map Operations | MO |
| Terminal Operations | Incorrect/Missing Terminal Operations | TO |
| Reduction Operations | Incorrect Reduction Operations | RO |
| Collector Operations | Incorrect/Missing Collector Operations | CO |
| Incorrect Action | Incorrect Action (e.g., $\lambda$-expression) | IA |

**Classifying Bug Fixes** Once bug fixes were identified, the authors studied the code changes to determine the category of bug fixes and whether the category relates to streams. Fortunately, we found that many commits reference bug reports or provide more details about the fix. Such information proved highly valuable in understanding the fixes. When in doubt, we also sent emails to developers for clarification purposes as git commits include email addresses.

## 6.2 Results

**Quantitative** Column **kws** of tab. 2 is the number of commits where occurrences of keywords were found and correspond to possible stream bug fixes. Column **exe** depicts the number of commits manually examined. From these 719 commits, we found 61 stream client code bug fixes. This is depicted in column **total** of tab. 4. Finding these bugs and understanding their relevance required a significant amount of manual labor that may not be feasible in more larger-scale, automated studies. Nevertheless, as streams become more popular (they were only introduced in 2014), we expect the usage and number of bugs related to streams to grow.

From the manual changes, we devised a set of common problem categories. Fixes were then grouped into these categories as shown in fig. 2 and tab. 4. A category legend appears in tab. 3, where column **name** is the “short” name of the bug category and is used in fig. 2. Column **description** is the categories extended name and column **acronym** is used in tab. 4.

Fig. 2 presents a hierarchical categorization of the 61 stream-related bug fixes. Bugs are represented by their category name (column **name** in tab. 3) and their bug counts. Categories with no count are *abstract*, i.e., those grouping categories.

Bugs are separated into two top-level categories, namely, bugs specifically related to stream API usage (stream-specific) and those tangentially related, i.e., bugs appearing in stream contexts but not specifically having to do with streams (generic). Generic bugs were further categorized into related to exception handling (EH), bounds checking (BC), poor performance (PP), and “other.” Generic exception handling bugs (6) include those where, e.g., $\lambda$-expressions passed to stream operations threw exceptions that were not handled properly. Generic bounds checking bugs (2) included those where $\lambda$-expressions missed traversal boundary checks, and generic performance bugs (2) were those involving,

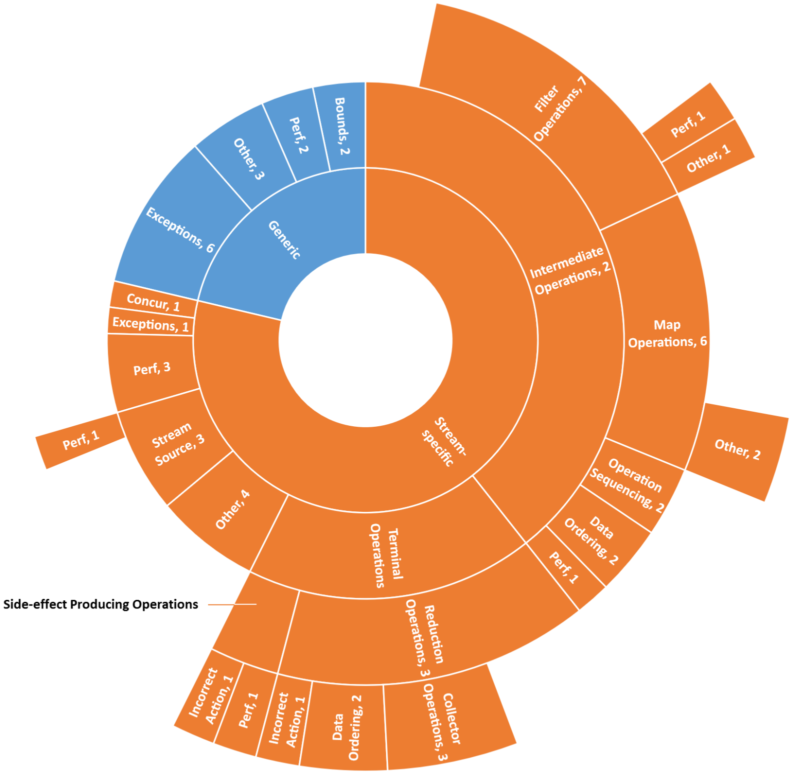

**Fig. 2.** Studied stream bugs and patches (hierarchical).

e.g., local variables holding stream computation results. The "other" category (3) is aligned with a similar one used by Tian and Ray [50] and involved syntactic corrections, e.g., incorrect types, and refactorings. Generally, "other" bugs can either be stream-specific or generic.

Stream-specific bugs are further divided into several categories corresponding to whether they involved intermediate operations (IO), terminal operations (TO), the stream source (SS), concurrency (CI), and performance and exception handling bugs specific to streams. IO-specific bugs (2) are related to intermediate operations other than filter operations (FO, 7) and map operations (MO, 6), e.g., `distinct()`. IO bugs are additionally partitioned into those involving incorrect operation sequencing (OS, 2), e.g., `map()` before `filter()`, data ordering (DO, 2), e.g., operating on a stream that should have been sorted, and performance bugs appearing in intermediate operations other than `map()` and `filter()` (1).

Terminal operations are split into two categories, namely, reduction operations (RO), e.g., `collect()`, `reduce()`, and side-effect producing operations, e.g., `forEach()`, `iterator()`. RO-specific bugs (3) were those related to scalar reductions, e.g., `anyMatch()`, `allMatch()`. RO-specific bugs related to collector operations (CO, 3), on the other hand, involve non-scalar reductions, e.g., a collector malfunction. RO-specific data ordering bugs (DO, 2) correspond to ordering of data related to scalar reductions, e.g., using `findAny()` instead of

**Table 4.** Studied stream bugs and patches (nonhierarchical).

| subject | BC | CI | CO | DO | EH | FO | IA | IO | MO | OS | PP | RO | SS | Other | Total |
|---|---|---|---|---|---|---|---|---|---|---|---|---|---|---|---|
| binnavi | | | | | | | | | | | | | | 1 | 1 |
| blueocean-plugin | | | | | | | | | | | | | | 1 | 1 |
| bootique | | | | | | | | | | | 1 | | | | 1 |
| che | | | | | 1 | 1 | | | 1 | | | 1 | | | 4 |
| cryptomator | 1 | | | | 2 | | | | | | 1 | | | 2 | 6 |
| dari | | | | | | | | | | | | | | 2 | 2 |
| eclipse.jdt.core | | | | | | | | | | | | | | 1 | 1 |
| eclipse.jdt.ui | | | | | | | | | | | | | 1 | | 1 |
| error-prone | | | | | 2 | 1 | 1 | | 3 | | 1 | 1 | 2 | 1 | 12 |
| guava | | | | | | | | | | | | | | 1 | 1 |
| JacpFX | | 1 | | 1 | | | | | | | 2 | | | | 4 |
| jdp | | | | | | | | | | | 1 | | | | 1 |
| jetty | | | 1 | 2 | | | | | 1 | | 3 | | | | 7 |
| jOOQ | | | | | | | | | 1 | | | | | | 1 |
| selenium | | | 2 | 1 | 2 | 5 | 1 | 2 | | 2 | | 1 | | 1 | 17 |
| threeten-extra | 1 | | | | | | | | | | | | | | 1 |
| Total | 2 | 1 | 3 | 4 | 7 | 7 | 2 | 2 | 6 | 2 | 9 | 3 | 3 | 10 | 61 |

`findFirst()`. RO-specific incorrect actions (IA, 1) is where there is a problematic $\lambda$ in a scalar reduction, e.g., an incorrect predicate in `noneMatch()`. Side-effect producing operation bugs also include incorrect actions (IA, 1), e.g., a problematic $\lambda$ in `forEach()`. Such operations can also exhibit poor performance (PO, 1).

Some bug categories are crosscutting, appearing under *multiple* categories. An example is performance. For this reason, tab. 4 portrays a nonhierarchical view of fig. 2, which is also broken down by **subject**, including a column for each bug category regardless of its parent category (acronyms correspond to tab. 3).

***Finding 7***: Bugs, e.g., performance, *crosscut* concerns, affecting multiple categories, both specifically and tangentially, associated with streams.

Performance issues dominate the functional (excluding "other") bugs depicted in tab. 4, making up the categories "Performance/API misuse" and "Performance," accounting for 14.75% (9/61) of the bugs found. While some of these fixes were more cleaning-based (e.g., superfluous operations), others affected central parts of the system and were found during performance regression testing [56].

***Finding 8***: Although streams feature performance improving parallelism, developers tend to struggle with using streams efficiently.

Despite widespread performance issues, concurrency issues (CI), on the other hand, were not prevalent (1.64%). The one concurrency bug was where a stream operation involved non-atomic variable access, which resulted in improper initialization [34]. Given that such a variable is accessed in a stream operation, however, it does indicate a possible side-effect and a need to consider refactoring such accesses to remove side-effects. This would make streams more amenable to efficient parallelization and perhaps promote more usage of parallel streams.

***Finding 9***: Concurrency issues were the *least* common streams bugs. However, concurrent variable access can cause thread contention, motivating future refactoring approaches that may promote more parallel streams.

The subjects selenium and error-prone had the most stream bugs with 27.87% and 19.67%, respectively. We hypothesize that this is due to the relatively large size of these projects, as well as their high usage of streams. Specifically, they fell into the top ten in terms of KLOC and stream method calls in tab. 2 and fig. 1, respectively, with ∼400 combined KLOC and 1,414 combined calls. Naturally, projects that use streams more are likely to have more bugs involving streams.

**Qualitative** We highlight several of the most common bug categories with examples, summarize common fixes, and propose preliminary best practices (**BP**) and anti-patterns (**AP**). Due to space limitations, only a single example of each BP/AP is shown; a complete set is available in our dataset [25]. Although some APs may seem applicable beyond streams, e.g., avoiding superfluous operations, we conjecture that streams are more prone to such patterns, e.g., due to the ease in which operations can be chained and the deferred execution they offer.

*SS→PP* Performance issues dominated the number of stream bugs found and also crosscut multiple categories. Consider the following performance regression [56]:

Project: jetty
Commit ID: 70311fe98787ffb8a74ad296c9dd2ba9ac431c9c
Log: Issue #3681

```
- List<HttpField> cookies = preserveCookies ? _fields.stream().filter(f ->
-     f.getHeader() == HttpHeader.SET_COOKIE).collect(Collectors.toList()) : null;
+ List<HttpField> cookies = preserveCookies?_fields.getFields(HttpHeader.SET_COOKIE):null;
```

The stream field is replaced with `getFields()`, which performs an iterative traversal, effectively replacing streams with iteration. The developer found that using iteration was faster than using streams [57] and wanted more "JIT-friendly" code. The developer further admitted that using streams can make code more easy to read but can also be associated with "allocation/complexity cost [55]."

***BP1***: Use performance regression testing to verify that streams in *critical* code paths perform efficiently.

In the following, a pair of superfluous operations are removed:

Project: JacpFX
Commit ID: 4f0d62d3a0987e47a4cbdf8e056bdf89713e6aac
Log: fixed class scanning

```
     final Stream<String> componentIds = CommonUtil
         .getStringStreamFromArray(annotation.perspectives());
     final Stream<Injectable> perspectiveHandlerList =
-          componentIds.parallel().sequential().map(this::mapToInjectable);
+          componentIds.map(this::mapToInjectable);
```

`getStringStreaFromArray()` returns a sequential stream, which is then converted to `parallel` and then to `sequential`. The superfluous operations are then removed.

***AP1***: Avoid superfluous intermediate operations.

*Fix:* Generally, fixes for performance problems varied widely. They ranged from replacing stream code with iterative code, as seen above, to removing operations, to changing the stream source representations. Depending on context, the bugs' effect can be either innocuous and cause server performance degradation.

*SS→TO→RO→CO* The stream API provides several ready-made `Collectors` for convenience. However, the API does not guarantee a *specific* non-scalar used during the reduction. On one hand, this is convenient as developers may not need a specific collection type; on the other hand, however, developers must be careful to ensure that the specific subclass returned by the API meets their needs.

In the following, the developer does not realize, until an incorrect program output, that the `Map` returned by `Collectors.toMap()` does not support nulls:

```
        Project: selenium
      Commit ID: 91eb004d230d8d78ec97180e66bcc7055b16130f
            Log: Fix wrapping of maps with null values. Fixes #3380
1 if (result instanceof Map) {
2 -  return ((Map<String, Object>) result).entrySet().stream().collect(Collectors.toMap(
3 -    e -> e.getKey(), e -> wrapResult(e.getValue())));
4 +  return ((Map<String, Object>) result).entrySet().stream().collect(HashMap::new,
5 +    (m, e) -> m.put(e.getKey(), e.getValue()), Map::putAll);
```

The ready-made collector (line 2) is replaced with a direct call to `collect()` with a particular `Map` implementation specified (line 4), i.e., `HashMap`.

> ***BP2***: Use collectors *only* if client code is *agnostic* to particular container implementations. Otherwise, use the *direct* form of `collect()`.

*Fix:* Collector-related bugs are typically corrected by not using a `Collector` (as above), changing the `Collector` used, or altering the `Collector` arguments. They often adversely affect program behavior but are also caught by unit tests.

*SS→IO* In the ensuing commit, `distinct()` is called on a concatenated stream to ensure that no duplicates are created as a result of the concatenation:

```
        Project: selenium
      Commit ID: eb7d9bf9cea19b8bc1759c4de1eb495829489cbe
            Log: Fix tests failing because of ProtocolHandshake
-    return Stream.concat(fromOss, fromW3c);
+    return Stream.concat(fromOss, fromW3c).distinct();
```

> ***BP3***: Ensure concatenated streams have distinct elements.

*Fix*: SS→IO bugs tend to be fixed by adding *additional* operations.

*SS→IO→Other* Developers "bridged" back to an imperative-style performed an operation, then switched back to streams to continue a more functional-style:

```
        Project: jetty
      Commit ID: 91e9e7b76a08b776be21560d7ba20f9bfd943f04
            Log: Issue #984 Improve module listing
1 -   List<String> ordered = _modules.stream()
2 -       .map(m->{return m.getName();}).collect(Collectors.toList());
3 -   Collections.sort(ordered);
4 -   ordered.stream().map(n->{return get(n);}).forEach(module->
5 +   _modules.stream().filter(m->...).sorted().forEach(module->
```

Each module is `mapped` to its name and `collected` into a list. Then, `ordered` is sorted via a non-stream `Collections` API. Another stream is then derived from `ordered` to perform further operations. However, on line 5, the bridge to a collection and subsequent sort operation is removed, and the computation remains within the stream API. It is now more amenable to parallelization.

> ***AP2***: Avoid "bridging" between stream API and legacy collection APIs.

Using a long λ-expression in a single `map()` operation may make stream code less "functional," more difficult to read [29], and less amenable to parallelism. Consider the abbreviated commit below that returns the occupied drive letters on Windows systems by `collecting` the first uppercase character of the path:

```
        Project: cryptomator
      Commit ID: b691e374eb2dad0284e13927e7c3fc1fdccae9bf
            Log: fixes #74
- return rootDirs.stream().map(path -> path.toString().toUpperCase()
-     .charAt(0)).collect(toSet());
+ return rootDirs.stream().map(Path::toString).map(CharUtils::toChar)
+     .map(Character::toUpperCase).collect(toSet());
```

The λ-expression has been replaced with method references, however, there are more subtle yet import changes. Firstly, as `CharUtils.toChar()` returns the first character of a `String`, there is a small performance improvement as the entire string is no longer turned to uppercase but rather only the first character. Also, the new version is written in more of a functional-style by replacing the single λ-expression passed to `map()` with multiple `map()` operations. How data is transformed in the pipeline is easily visible, and future data transformations can be easily integrated by simply adding operations.

***AP3***: Avoid too many operations within a single `map()` operation.

*Fix*: "Other" non-type correcting fixes, e.g., refactorings, included introducing streams, sometimes from formerly iterative code (3), replacing `map()` with `mapToInt()` [20], and dividing "larger" operations into smaller ones.

## 7 Threats to Validity

Subjects may not be representative. To mitigate this, subjects were chosen from diverse domains and sizes. They have also been used in previous studies (e.g., [20,22]). Although java-design-patterns is artificial, it is a reference implementation similar to that of JHotDraw, which has been studied extensively (e.g., [31]). Also, as streams are relatively new, we expect a larger selection of subjects as they grow in popularity.

Entry points may not be correct, which could affect how stream attributes are calculated. Since standard entry points were chosen, these represent a superset of practically true entry points. Furthermore, there may be custom streams or collectors outside the standard API that we are not considered. As we aim to understand stream usage and misuse in the large, we hypothesize that the vast majority of projects using streams use ones from the standard libraries.

Our study involved many hours of manual validation, which can be subject to bias. However, we investigated referenced bug reports and other comments from developers to help us understand changes more fully. We also reached out to several developers via email correspondence when in doubt. All but one returned the correspondence. The NLP features of `gitcproc` may have missed changesets that were indeed bug fixes. Nevertheless, we were still able to find 61 bugs that contributed to a rich bug categorization, best practices, and anti-patterns. Furthermore, `gitcproc` has been used previously in other studies.

## 8 Related Work

Previous studies [29,32,36,46,51] have focused specifically on λ-expressions. While λ-expressions are used as arguments to stream operations, our focus is on stream *operations* themselves. Such operations transition streams to different states, which can be detrimental to parallel performance [24,35]. Also, since streams can be aliased, we use a tool [24] based on typestate analysis to obtain stream attributes more reliably than AST-based approaches. We also study bugs related to stream usage and present developer feedback—fixing bugs related to streams may not involve changing λ-expressions; bugs can be caused by, e.g., an incorrect sequence of stream operations. Lastly, although Nielebock et al. [36] consider λ-expressions in "concurrency contexts," such contexts do not include streams, where λ-expressions can easily execute in parallel with minimal syntactical effort.

Khatchadourian et al. [24] report on some stream characteristics as part of their refactoring evaluation but do so on a much smaller-scale, as their focus was on the refactoring algorithm. The work presented here goes significantly above in beyond by reporting on a richer set of stream characteristics (e.g., execution mode, ordering), with a noteworthy larger and updated corpus. We also include a comprehensive categorization of stream-related bug fixes, with 719 commits *manually* analyzed. Preliminary best practices and anti-patterns are also proposed.

Zhou et al. [60] conduct an empirical study on 210 service quality issues of a big data platform at Microsoft to understand their common symptoms, causes, and mitigations. They identify hardware faults, systems, and customer side effects as major causes of quality issues. There are also empirical studies on data-parallel programs. Kavulya et al. [19] study failures in MapReduce programs. Jin et al. [18] study performance slowdowns caused by system side inefficiencies. Xiao et al. [58] conduct a study on commutativity, nondeterminism, and correctness of data-parallel programs, revealing that non-commutative reductions lead to bugs. Though related, our work specifically focuses on stream APIs as a language feature and programming paradigm, which pose special considerations due to its shared memory model, i.e., interactions between the operations and local memory. Bloch [6, Ch. 7] also puts-forth stream best practices and anti-patterns. However, ours are based on a statistical analysis of real-world software and first-hand interactions with real-world developers.

Others also study language features. Parnin et al. [43] study the adoption of Java generics. Dyer et al. [11] build an expansive infrastructure for studying the use of language features over time. Khatchadourian and Masuhara [22] employ a proactive approach in empirically assessing new language features and present a case study on default methods. There are also many studies regarding bug analysis. For example, Engler et al. [13] present a general approach to inferring errors in systems code, and Tian and Ray [50] study error handling bugs in C.

## 9 Conclusion & Future Work

This study advances our understanding of stream usage and bug patterns. We have surveyed common stream operations, attributes, and bugs specific and tangentially related to streams. A hierarchical taxonomy of stream bugs was devised, preliminary best practices and anti-patterns were proposed, and first-hand developer interactions were detailed. In the future, we will explore stream creation, use our findings to devise automated error checkers, and explore topics that interest stream developers. Lastly, we will investigate applicability to other streaming frameworks and languages.

**Acknowledgments** We would like to thank Krishna Desai and Robert Dyer for work on data summarization and discussions, respectively. Support for this project was provided by PSC-CUNY Award #617930049, jointly funded by The Professional Staff Congress and The City University of New York. This material is based upon work supported by the National Science Foundation under Grant No. CCF 1845893, CNS 1842456, and CCF 1822965.
